# Navigating constrained training participation: a grounded theory of training avoidance among Chinese college football student-athletes

**DOI:** 10.3389/fpsyg.2026.1904154

**Published:** 2026-08-03

**Authors:** Yangjia Xiong, Minghan Li, Xingyuan Song, Ying Chen

**Affiliations:** 1 College of Physical Education, Huaqiao University, Quanzhou, Fujian Province, China; 2 College of Physical Education, Yunnan Minzu University, Kunming, Yunnan Province, China

**Keywords:** institutional constraints, grounded theory, college sport, training avoidance, athlete training disengagement

## Abstract

**Introduction:**

Training avoidance in sport is often interpreted through a deficit lens, yet little is known about how athletes regulate participation when leaving a team is institutionally costly. This study examined training avoidance within Chinese college football as a form of constrained participation.

**Methods:**

Semi-structured interviews were conducted with 16 Chinese college football student-athletes, all admitted through the Sports Special Admission policy. Data were analyzed using Straussian grounded theory procedures, including iterative theoretical sampling, constant comparison, open, axial, and selective coding, and negative-case analysis.

**Results:**

The analysis generated Navigating Constrained Training Participation as the core category. Four causal conditions shaped training avoidance: Status Marginalization Pressure, Inter-Role Conflict, Informal Power Oppression, and Asymmetrical Rule Enforcement. Athletes responded through a flexible, context-dependent repertoire of Covert Energy Conservation, Psychological Defense, Legitimation Maneuvering, and Overt Resistance. These strategies were not a fixed progression; athletes combined, revisited, or abandoned them as perceived risk, bodily capacity, available justifications, and coach relations changed. The process provided short-term protection while potentially contributing to longer-term erosion of skill, belonging, and participation.

**Discussion:**

Training avoidance is better understood not as an individual moral deficit but as a systemically produced and structurally conditioned adaptation. The model clarifies how athletes exercise constrained agency and offers a transferable explanation for sport-education systems in which exit is costly and participation is tied to educational opportunities, grading, resources, or status.

## 1 Introduction

Student-athletes sometimes remain formally attached to teams while reducing their physical effort, psychological investment, attendance, or exposure to evaluation. Although such conduct is widespread, it has remained at the margins of scholarly attention. In China, the sport-education integration agenda emphasizes student-athlete training participation (Li, 2024; Shi et al., 2024), yet reduced engagement remains pronounced in college football teams (Yang, 2022; Li et al., 2022). These actions occupy the space between full engagement and complete exit and range from concealed reductions in effort to openly visible resistance.

This space is especially consequential in Chinese collegiate football. All participants in this study entered higher education through the Sports Special Admission policy (SSA; tiyudanzhao), and their academic progression, specialized football courses, team membership, and relationships with coach-instructors can become closely intertwined (General Administration of Sport of China, Ministry of Education, 2024). Leaving is therefore not a simple voluntary decision. Even where no formal rule directly binds enrollment to attendance, the overlap between coaching and academic evaluation can raise the perceived cost of withdrawal and constrain athletes' available responses.

Existing research has examined related phenomena, including fear-avoidance (Dover and Amar, 2015), athlete burnout (Raedeke and Smith, 2001), and sport withdrawal and disengagement (Stavitz et al., 2025; Eliasson and Johansson, 2021; Battaglia et al., 2024). These concepts are relevant but not interchangeable with training avoidance. Burnout denotes a psychological syndrome associated with chronic stress, disengagement commonly describes a broader reduction in involvement, and withdrawal usually culminates in leaving sport. Training avoidance, as examined here, concerns situated actions through which athletes regulate expected participation while continuing to occupy a formal place in the team. It may coexist with burnout or contribute to disengagement, but neither condition is necessary for avoidance to occur.

This study addresses the unresolved question of how athletes act when full compliance is difficult but exit remains costly. It adopts a non-moralizing analytical position: reduced training engagement is not presumed to indicate laziness, weak commitment, or defective character. Drawing on in-depth interviews with 16 participants, the study conceptualizes training avoidance as context-responsive efforts to reduce, redirect, conceal, justify, or resist expected training engagement while remaining formally located within the sport-education system. The contribution lies not simply in classifying behaviors from covert to overt, but in explaining how structurally conditioned pressures, athlete judgments, action/interaction strategies, and consequences are integrated through constrained agency. Chinese collegiate football is treated here as a high-visibility case of constrained sport-education participation rather than as an exceptional national case. The resulting model provides sensitizing concepts for comparison with dual-career academies, scholarship-based university sport, and other systems in which educational access, selection, or coach authority makes exit costly; future research can examine which conditions and strategy configurations travel across these settings and where contextual revision is required. Straussian grounded theory methodology was adopted (Corbin and Strauss, 2015) because it supports theory construction around conditions, actions/interactions, and consequences while preserving participants' meanings. The research questions were: (1) How do Chinese college football student-athletes understand and experience training avoidance? (2) How do institutional conditions shape the strategies through which they navigate constrained training participation, and what consequences follow?

## 2 Methods

### 2.1 Grounded theory methodology

This study adopts Straussian grounded theory methodology (GTM; Corbin and Strauss, 2015). GTM is suited to explaining actions and interactions in areas where existing concepts do not adequately account for the process under investigation. A defining feature is theoretical sensitivity: the researcher's capacity to recognize analytically significant variation and relationships in the data (Glaser and Strauss, 1967). In this study, familiarity with Chinese collegiate sport supported attention to training routines, institutional constraints, and athlete language, while constant comparison and reflexive memoing were used to prevent contextual familiarity from substituting for empirical evidence. The objective was not to force participants into a predetermined engagement continuum, but to construct an explanatory account of how training participation was negotiated under changing conditions.

### 2.2 Researchers' philosophical perspective and positionality

This study works within a pragmatist framework consistent with Straussian GTM (Corbin and Strauss, 2015). Pragmatism treats action as situated and consequential: people interpret conditions, act within available possibilities, and revise their conduct in response to consequences. Training avoidance was therefore approached neither as a fixed trait nor as an unconstrained choice, but as action formed through the relationship between athletes and their sport-education environment. Pragmatist epistemology also recognizes knowledge as co-constructed through researcher-participant interaction (Battaglia et al., 2022; Kelly and Cordeiro, 2020). The analytical task was to explain how participants understood constraints, assessed risks, and adjusted training participation, while remaining attentive to the institutional conditions that bounded those adjustments.

Researcher positionality feeds into theoretical sensitivity. Examining it helps reveal how the researcher's identity and experiential background enter and shape the research process. The first author conceived and led this study, with a second coder brought in during the early stages of coding to strengthen analytical rigor. The first author's first-hand experience as a former college team member supplied a deep, lived understanding of Chinese collegiate football training culture, coach power structures, and academic-training conflicts. It also opened the door to pre-existing trust with some participants, which helped them speak more candidly about sensitive training experiences. While the insider status was conducive to building rapport, participants were reminded up front that nothing they said would reach coaches or teammates. The point was their genuine experience, not the “correct” answer.

The insider position brought two clear strengths. One, data collection benefited from the researcher's contextual and cultural familiarity with the group, helping participants open up about their real training experiences rather than slipping into performative accounts (Dwyer and Buckle, 2009). Two, during analysis, the researcher's embodied experience and contextual knowledge of training situations enabled a more sensitive identification of layers of implicit meaning and subtle nuance in the data, considerably sharpening theoretical sensitivity during open and axial coding (Glaser and Strauss, 1967).

Being an intimate insider, however, also carries clear risks. The line between preconceptions rooted in personal training experience and what participants were actually saying could blur, allowing personal experience to substitute for data (Taylor, 2011). To manage this, reflexive practice ran throughout data collection and analysis. Before each interview, preparation notes were used to keep questions open-ended. Immediately after each interview, memos captured emotional reactions and analytical hunches, so these could later be flagged and set apart from concepts genuinely emerging from the data. During coding, priority was given to participants' *in vivo* language when naming concepts, and the question “Does this judgment come from the data or from my own experience?” was regularly applied as a self-audit for category assignment (Ellis, 2007; Etherington, 2007).

Trustworthiness was built through the second coder's involvement during initial coding, the first author's sustained critical reflexivity, and a commitment to systematic transparency throughout the analytical process.

### 2.3 Participants and theoretical sampling

Sixteen nationally certified competitive student-athletes were recruited, all of whom were currently or previously registered with Chinese university football teams (see Table 1). For this study, nationally certified competitive student-athlete was an operational, study-specific designation: each participant held a National Level II Athlete title or above in football, indicating certified competitive experience based on performance in officially recognized events. The designation does not imply professional athlete status or refer exclusively to the national high-level sports-team admission category. All 16 participants entered university through the Sports Special Admission (SSA) pathway. The theoretical sampling design, following (Corbin and Strauss, 2015), was set up to capture broad variation in training avoidance experiences and multiple vantage points within Chinese college football. Undergraduates (*n* = 12) provided accounts of training avoidance as part of everyday training life, while postgraduates (*n* = 4, including one doctoral student) offered retrospective and cross-stage perspectives. Male athletes (*n* = 12) made up the majority of the sample, consistent with the gender profile of Chinese college football teams. Female athletes (*n* = 4) were recruited to explore how gendered bodily and institutional conditions might shape training participation.

**Table 1:** Overview of participants.

Participant	Interview (min)	Gender	Educational stage	Primary position	University team tenure (years)
P01	30.32	Female	Senior	Defensive midfielder	4
P02	25.75	Male	Junior	Forward	3
P03	43.00	Male	Sophomore	Forward	2
P04	46.05	Male	Junior	Central midfielder	3
P05	37.25	Male	First year	Central midfielder	1
P06	35.88	Female	Master's year 2	Center back	2
P07	42.92	Female	Doctoral year 1	Attacking midfielder	5
P08	41.10	Male	Master's year 1	Goalkeeper	2
P09	51.00	Male	Senior	Defensive midfielder	4
P10	30.08	Female	Junior	Central midfielder	3
P11	37.38	Male	First year	Center back	< 1
P12	28.38	Male	Junior	Attacking midfielder	3
P13	31.57	Male	Postgraduate	Utility player	4
P14	37.37	Male	First year	Center back	1
P15	24.50	Male	Senior	Central midfielder	4
P16	32.90	Male	Junior	Center back	3

University team tenure refers only to participation after university enrollment and is not equivalent to total football involvement or years of systematic training. A value of < 1 indicates one completed university semester.

No predetermined numerical threshold was imposed for university-team tenure. Eligibility instead required the certification described above and first-hand experience of university-team training sufficient to provide concrete accounts of recurring training practices. University-team tenure was used as a dimension of theoretical sampling rather than as an inclusion cutoff.

To ensure breadth of perspective, participants were recruited across playing positions and relative team positions. Leadership responsibilities were emergent background characteristics rather than separate recruitment strata. P07 reported concurrent experience as a player and campus-team coach; this dual role emerged during the interview rather than through a separate recruitment route, and no predetermined percentage-of-time threshold was used to classify a participant as a player-coach. P07 was included and analyzed primarily in relation to the student-athlete role, while the coaching experience was treated as contextual background. P09 was a team captain, and P13 later assumed deputy captain and informal peer-coaching responsibilities, including consultation on lineups and assistance with training. Prior football backgrounds also varied: some participants had provincial-team, professional-club academy, overseas, or intensive school-team experience, whereas others had primarily university-team backgrounds. No participant reported systematic competitive training in another sport; occasional exposure to other sports was treated as background experience rather than as a separate competitive training history.

Team status was treated as a relational and potentially changing analytical position rather than a formal administrative rank. It was derived from participants' accounts of match selection, playing time, starter-substitute status, positional stability, coach recognition, captaincy, and informal leadership responsibilities. Comparisons therefore focused on relative core, regular, and marginal positions while recognizing that an athlete's position could change over time.

Participants generally reported long-term involvement in football, but football involvement, systematic training, and university team tenure were not treated as equivalent measures. For example, some described 9–15 or more years of football participation, whereas P14 distinguished 17–18 years of involvement from approximately 4–5 years of systematic training. Because total training duration was not elicited uniformly from every participant, it was used as a qualitative comparison dimension rather than a quantitative covariate.

In line with the study's focus on athletes' subjective experiences and meaning-making processes, the sample was purposively limited to student-athletes. Coaches, team managers, and institutional administrators were not recruited. This decision rested primarily on the study's aim to construct a conceptual definition and classification of training avoidance behavior from the standpoint of those who enact it, the student-athletes themselves, rather than through the evaluative lens of observers or authority figures. Moreover, the structural features of the Chinese college sport system, particularly the SSA policy, create a distinctive power asymmetry in coach-athlete relationships. Coaches hold considerable authority over athletes' institutional lives, and including them as co-participants risked conflating two structurally different perspectives, undercutting the conceptual clarity of an athlete-centered analysis. Participants were drawn from multiple universities across northern and southern China, to capture institutional diversity in training culture and in how the SSA policy is implemented on the ground.

**Figure 1:**
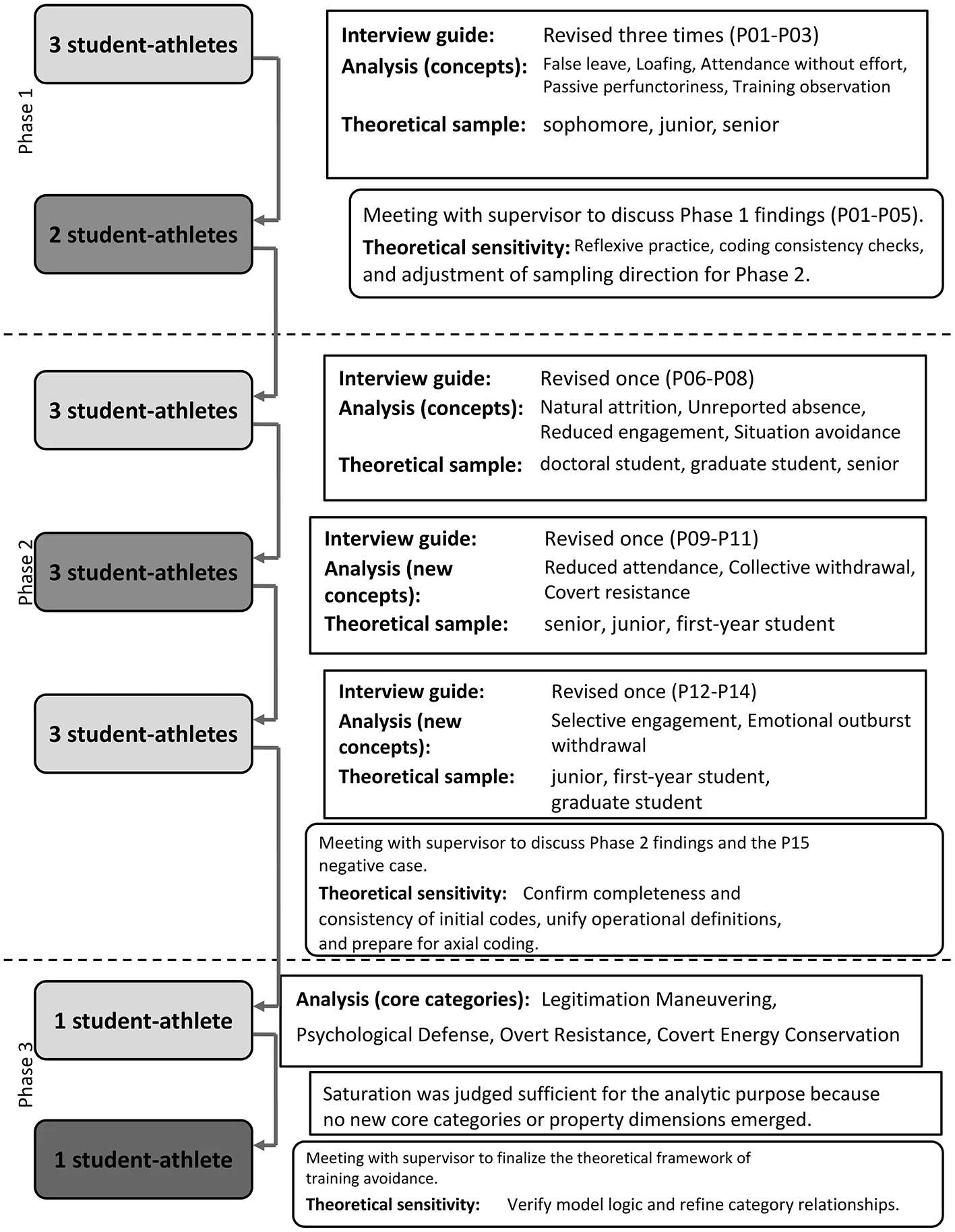
Theoretical sampling process and interaction between data collection and analysis.

Participant recruitment proceeded in three phases (see Figure 1).

Phase 1. Five student-athletes were interviewed, spanning first-year through senior undergraduate years. The main task at this stage was to establish that training avoidance behavior was real and observable. Beyond gathering the interviewees' own experiences, focused questions were asked about what they had seen among teammates, confirming that the phenomenon was both common and varied in texture across daily training. Initial analysis surfaced core dimensions such as academic-training conflict, physical fatigue, and coach-related factors, which laid the groundwork for the semi-structured interview guide used in later phases.

Phase 2. Because Phase 1 linked academic demands, team status, and coach-related pressures to reduced participation, nine additional participants were recruited, including two master's students and one doctoral student. Sampling followed the developing categories: participants with contrasting status positions, role obligations, and training histories were sought to clarify when similar pressures produced different responses. Ongoing open and axial coding distinguished causal conditions from action/interaction strategies and identified variation in strategy visibility, domain of withdrawal, need for justification, and institutional risk. By the 14th interview, three consecutive interviews had produced no substantively new categories, properties, or category relationships, indicating preliminary theoretical saturation.

Phase 3. Discriminant sampling was used to test category boundaries and potential counter-examples (Corbin and Strauss, 2015). P15 had moved from a substitute position to a regular starting role but reported little change in his general training orientation after the status transition. This negative case challenged a deterministic interpretation in which improved status necessarily increases engagement. P16 reported fatigue under higher training loads but did not describe a wish to avoid training, providing a second contrast between physical strain and enacted avoidance. Review of P15 and P16 by both coders indicated that these cases refined category boundaries without adding new core categories or property dimensions. On this basis, saturation was judged sufficient for the analytic purpose rather than formally confirmed.

## 3 Data collection

### 3.1 Individual semi-structured interviews

Before the first interview, the study protocol was reviewed by the Academic Committee of the College of Physical Education, Huaqiao University, which determined that the study qualified for exemption from full ethics review under institutional guidelines because it involved low-risk, non-invasive social science research using semi-structured interviews with adult participants. Interview guides were constructed as flexible, open-ended instruments following Rubin and Rubin's (Rubin and Rubin, 2012) responsive interviewing model, in which questions are designed to pursue the meaning behind participants' accounts rather than to confirm predetermined categories. Consistent with the iterative logic of grounded theory methodology, the interview guides were revised three times in Phase 1 and three times in Phase 2, with newly emerged concepts continuously directing the direction of subsequent data collection.

The study employed face-to-face interviews (*n* = 9) alongside telephone interviews (*n* = 7). Informed consent forms were provided for all interviews, and consent was obtained from all interview participants. Interview duration in Phase 1 ranged from 26 to 46 min, with a mean duration of 36 min (SD = 8.48); in Phase 2, duration ranged from 28 to 51 min, with a mean of 37 min (SD = 7.07); in Phase 3, duration ranged from 25 to 33 min, with a mean of 29 min (SD = 5.94). Phase 2 had the longest mean interview duration because this phase included in-depth interview samples such as team captains and participants with professional-club academy backgrounds, while Phase 3, as confirmatory interviews, showed a marked reduction in duration, consistent with the increased interview focus after preliminary theoretical saturation. A total of 16 semi-structured interviews were completed, with an overall mean duration of 36 min (SD = 7.89). Interview duration is reported descriptively and was not treated as an indicator of interview quality or analytical rigor.

### 3.2 Data analysis

All interviews were audio-recorded in full and transcribed verbatim to ensure data fidelity. Analysis followed the flexible strategy of Straussian GTM. All coding was facilitated by NVivo 14, which supported the systematic management of the coding scheme and the iterative development of node hierarchies. NVivo 14 was used solely for data management and manual coding organization, not for automated coding.

Once transcription was complete, recordings were checked repeatedly against transcripts before coding began. Open coding used microanalysis and constant comparison to identify actions, meanings, conditions, and consequences in participants' accounts (Corbin and Strauss, 2015). The first two transcripts were independently coded by the first and second coders, who compared coding decisions, discussed their interpretations, and reached consensus on category boundaries; no coding disagreements were identified. The first author then coded the remaining transcripts, consulting the second coder when conceptual ambiguity arose. Axial coding related initial concepts to subcategories and main categories through the Straussian paradigm of conditions, central phenomenon, action/interaction strategies, and consequences. Comparisons were made within and across participants, including contrasting team status, educational stage, prior training background, and cases in which similar pressures did not produce the same strategy. For participants with leadership responsibilities, first-person accounts of their own participation were analyzed as athlete experiences, whereas evaluations of teammates were treated as role-positioned contextual data and compared with accounts from participants without leadership responsibilities.

Fatigue, stress, and burnout-related language were not automatically coded as training avoidance. They were treated as conditions or subjective states unless the account also described an action through which expected participation was reduced, concealed, redirected, justified, or resisted. Negative cases strengthened this distinction: P07 described reluctance to wake for early training but reported full engagement after attending; P16 reported fatigue without a wish to avoid training; and P10 described acute stress while continuing to train. The interviews were not used to diagnose athlete burnout.

Selective coding integrated the categories around a process capable of explaining both constrained conditions and variation in athlete action. The initial formulation, “Gradient Regulation of Training Engagement,” captured differences in the intensity, visibility, and risk of the four strategies, but did not fully integrate costly exit, institutional power, strategic combination, and feedback from consequences. Continued comparison of category relationships and integrative diagrams therefore refined the core category as “Navigating Constrained Training Participation.” This formulation linked four causal conditions, the central phenomenon of training avoidance as constrained participation, four flexible action/interaction strategies, and short- and long-term consequences. Gradient regulation was retained as a dimensional property of the process rather than a fixed sequence. Sixteen analytical memos, participant reflections on coding interpretations, negative-case analysis, and integrative diagrams supported category refinement and theoretical integration.

### 3.3 Evaluation of the methodology

Methodological consistency was evaluated against Corbin and Strauss's 16 criteria (11, pp. 350–351). First, sampling and data collection proceeded iteratively, with later participants selected to elaborate differences in team status, role obligations, prior training background, and strategy use until no important new categories, properties, or relationships emerged. Second, analytical grounding was maintained through open, axial, and selective coding; constant comparison; 16 analytical memos; negative-case examination; and integrative diagrams. Open coding generated 73 initial codes, and 36 representative coding chains with participant quotations are reported in the Supplementary material. Axial coding distinguished four causal conditions, the central phenomenon, four action/interaction strategies, and two consequence horizons. Selective coding tested which concept could integrate these components without imposing a mandatory sequence. P15's stable orientation across a substitute-to-starter transition and P16's fatigue without avoidance were retained as negative cases that bounded status and fatigue explanations. Third, reflexivity and validation were supported through a research journal, documentation of analytical decisions, participant reflections, and independent coding of early transcripts by a second coder. These procedures were used to refine conceptual boundaries and preserve an auditable path from participant accounts to category integration.

## 4 Results

**Figure 2:**
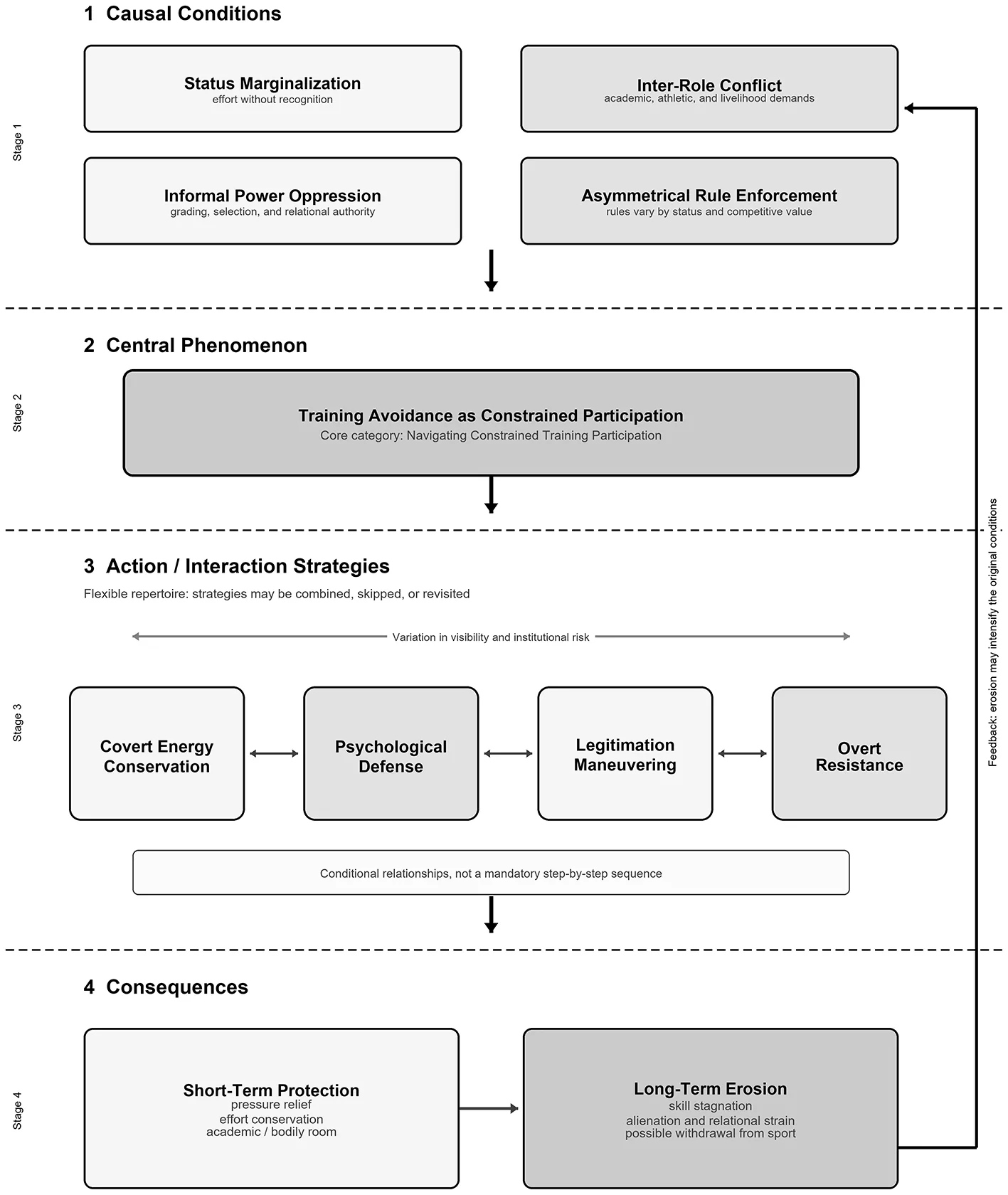
Paradigmatic model of training avoidance as constrained participation.

The findings are organized through the Straussian paradigm of causal conditions, central phenomenon, action/interaction strategies, and consequences. Figure 2 summarizes the relationships among these components. The arrows represent analytical connections and feedback, not a mandatory chronological sequence.

### 4.1 Causal conditions of training avoidance

Four interrelated conditions made training avoidance intelligible within Chinese collegiate football: Status Marginalization Pressure, Inter-Role Conflict, Informal Power Oppression, and Asymmetrical Rule Enforcement. These conditions operated in combination rather than as isolated predictors. They did not automatically produce avoidance; instead, they constrained athletes' available responses and made the regulation of participation a recurring practical problem.

#### 4.1.1 Status marginalization pressure

Athlete dropout has been linked directly to declining sport enjoyment and eroding interest, and the process tends to be gradual rather than a single definitive decision (Eliasson and Johansson, 2021).

“They don't feel valued in the team. No playing time, no position. Training just feels pointless, and after a while they stop showing up.” (P09)

“Some guys train for ages and still never make it into an official match. Zero sense of involvement. Of course a lot of them want to leave.” (P01)

In these accounts, a tension surfaces between “training for ages” and “zero sense of involvement.” The athletes are not failing to put in effort. Their efforts meet with no response. P12 confirmed the experience in the first person: “If I could just get into the rotation, I'd want to play. Right now I have zero chance. There's no way they're taking me to matches.” Taken together, these accounts point to something important: the essence of status marginalization lies not in rejecting training itself, but in an erosion of meaning around training participation. Athletes keep investing, yet no visible return comes from that investment.

“The coach picks players for matches purely on subjective whim. Gets the captain to help choose. Doesn't even understand the game himself.” (P04)

P04 had trained overseas in Spain and had experience in a professional youth academy. His assessment of the coach's professionalism was grounded in cross-contextual comparison, which lifts this criticism beyond the level of personal grievance. When the transparency and fairness of selection criteria come under question, athletes' investment in training loses its logic of anticipated return.

“A lot of substitute players never get a chance to play. Their performance gets worse and worse. Gradually they stop participating. They give up on themselves. Let themselves go.” (P13)

From P2's “zero chance” to P3's account of giving up on themselves and letting themselves go, one observed pathway links institutional marginalization with progressive behavioral disengagement. What the data reveal is that training avoidance behavior is not necessarily a rejection of training itself. It can reflect the erosion of meaning attached to training participation.

#### 4.1.2 Inter-role conflict

Inter-Role Conflict captures competing obligations attached to participants' positions as students, athletes, family members, and income earners. These conflicts were not merely scheduling inconveniences. Different roles carried unequal institutional penalties, and only some obligations were recognized as legitimate grounds for reducing or missing training.

Research has demonstrated that time conflicts between academic and athletic commitments represent one of the most commonly reported structural reasons for sport withdrawal among youth athletes (Crane and Temple, 2015).

“Specialized football class in the morning, team training in the afternoon… add it up, that's eight or nine sessions a week.” (P05)

For SSA student-athletes, specialized football courses and team training are typically scheduled back to back. Training twice a day becomes the norm. Under this arrangement, conflict is not an occasional accident. It is a rhythm built into the daily timetable.

“After training you‘re drenched in sweat, no time to shower or eat. By the time you rush to class you're completely wiped out, basically in a fog.” (P03)

The transition between training and class, then, is not recovery. It is moving from one kind of fatigue straight into another. As P11, a first-year student, recalled: “Eighteen of us enrolled as freshmen. Now only three are still training.” That attrition rate reflects something deeper than individual collapse of will. The institution itself is filtering out those who try to keep up.

“My master's thesis draft was due. There was no way I could juggle the research workload and the intensive training. So I just let go of all the training after that.” (P07)

What this confirms is that training gets sacrificed first not because it matters less, but because the consequences of missing one training session are lighter than the consequences of handing in an incomplete thesis. P13 recalled what a university instructor told him: “Our main job is being students, not professional athletes. You have to get your academics sorted first.” Even among those who are highly committed to training, “student first, athlete second” is an identity ranking that the institution repeatedly reinforces. But the real nature of this ranking lies in the asymmetry of institutional penalties. In practice, missing training can be forgiven by academic instructors. Missing coursework cannot be forgiven by the coach. And that can mean failing a course.

The institution is not entirely rigid. P13 described the accommodating arrangements made during exam weeks: six training sessions per week cut to two or three, signaling that those in charge do recognize the conflict and are willing to make temporary concessions at critical moments. Yet the very temporariness of the concession underscores the permanence of the conflict. If the system could resolve the conflict systematically, it would not need to ease academic pressure by temporarily scaling back training.

The legitimacy of an obligation therefore depended on whether the institution recognized the role from which it arose. Athletes had to negotiate not only which responsibility to prioritize, but also how to make that responsibility intelligible within the team's evaluative order.

“This competition is something we signed up for voluntarily, not something the university requires. It mainly comes down to the supervisor's priorities.” (P06)

“Official business, like a college meeting or work shift duties… that kind of leave request, I think is completely fine. Private matters… if you could have dealt with it in the morning but deliberately scheduled it during training time… that I can't accept. It's obvious you just don't want to train.” (P10)

So the rule turns out to be fairly straightforward. Only those institutional obligations that the institution itself formally recognizes, such as college meetings or official duties, can serve as valid grounds for missing training. A private matter, even a genuine one, does not make the cut if it could have been handled outside training hours. What matters is not whether the athlete really has something to do. What matters is whether that something comes from a role the institution already acknowledges.

The data from P08 opened up another possibility: “Taking private coaching jobs outside, substitute teaching, stuff like that, is about making money… whichever one you think brings you greater benefit, whichever one helps you more, that's what you‘ll choose.” Student-athletes do not only possess dual identities; they may also hold the alternative identity of “laborer,” that is, they take private coaching jobs outside or play in pay-for-play matches to earn money. For them, the “laborer” identity is equally real and equally carries obligations. This kind of reason may be highly reasonable within the athletes' own value ranking, but within the coach's evaluation system, it is usually not accepted as a legitimate reason for absence. This gap between “self-acknowledged but institutionally unacknowledged” forces athletes to fall back on other justification production strategies (such as physiological boundaries) or accept the reality that their absence will not be recognized.

#### 4.1.3 Informal power oppression

Under the SSA system, team coaches typically also serve as the instructors of student-athletes' specialized sport courses. This means coaches do not merely arrange training; they hold evaluative authority over athletes' course grades. Within the Chinese cultural context, athletes occupy a subordinate position and have long been accustomed to complying with coach demands while suppressing their genuine feelings. Coaches' authoritarian leadership style is widely accepted in Chinese college sport settings, and athletes display a relatively high level of cultural tolerance toward this style (Jin et al., 2022). What student-athletes face in training, then, extends beyond the coach's instructional commands. Hidden underneath is the reality that resisting, distancing oneself from, or abandoning training can come at the cost of course grades.

“The coach threatened us with failing grades, wouldn't let us go out for high-level competitions, held onto us like private property.” (P05)

The threat of course failure bites not because of the coach's personal authority, but because the coach genuinely wields legitimate grading power. P02 described a coach who developed an inexplicable negative attitude toward him: “Same training mistake. With that other player the coach might say nothing at all. With me, he'd bring it up again and again, chewing me out.” The consequence of differential treatment was not interpersonal friction. It was a kind of institutional helplessness. The student-athlete could not change the situation by adjusting his own behavior, because the evaluation criteria themselves were opaque.

“First day I got here, he wouldn't let me play. He hadn't even seen me on the court yet. I have no idea why.” (P12)

The coach's judgment did not need to be based on actual performance. It could take effect before the athlete had even demonstrated what he could do.

“Guys who play worse than me get on the court instead. I genuinely don't understand.” (P12)

When selection criteria become unpredictable and unintelligible, training investment loses its direction.

Negative emotional contagion within a team can undermine collective efficacy and team performance, and once internalized emotional expression norms are violated, dysfunctional emotional contagion may be triggered (Campo et al., 2019). Speaking from his vantage point as team captain, P09 noted that when teammates “have a conflict with the coach, they don't dare push back openly. So they engage in covert resistance, don't put in full effort at training, and it drags down the whole team's training quality.” He added that “one person's negativity can trigger a resonance, and everyone sinks into it.” The reason they dare not push back openly is clear: the coach's grading power makes the cost of open conflict too high. Grievances, then, get converted into disengaged effort during training and, through emotional interaction among teammates, diffuse outward into a collective phenomenon. This quiet sinking is harder for the institution to detect and correct than open conflict would be, and it erodes training more persistently.

#### 4.1.4 Asymmetrical rule enforcement

Asymmetrical Rule Enforcement describes the unequal application of attendance, selection, and disciplinary rules according to athletes' team status and competitive value. This condition shaped anticipated consequences and normalized violations when comparable conduct produced different responses for different players.

P14 put the status-contingent character of attendance enforcement plainly: “There's an attendance system, but some key players just don't show up, and the coach says nothing, because the matches need them. The ones who play badly also don't show up, and gradually they just stop playing.” The formal rule therefore existed without being applied universally. Competitive value operated as an informal exemption for core players, while the absence of marginal players could go unaddressed. The analytical issue here is not the act of absence itself, which is examined under Overt Resistance, but the unequal institutional response to comparable conduct. This asymmetry made consequences difficult to predict and shaped the risks athletes considered when regulating participation.

### 4.2 Central phenomenon: training avoidance as constrained participation

Across these conditions, the central phenomenon was Training Avoidance as Constrained Participation: a systemically produced and structurally conditioned process of adaptation through which student-athletes reduced, redirected, concealed, justified, or resisted expected training engagement while remaining formally attached to the sport-education system. This formulation does not treat training avoidance as evidence of an individual moral deficit, nor does it imply that the behavior or its institutional conditions are neutral. Instead, it directs attention to how athletes enacted constrained agency under costly exit, unequal authority, competing role obligations, and asymmetrical rule enforcement.

P09 described training avoidance as “a perfectly normal phenomenon” and “neutral.” We interpret this not as evidence that training avoidance or its institutional conditions were neutral, but as resistance to moralizing labels such as laziness, fear, or personal failure. P3's comparison with students zoning out and employees coasting, together with P5's description of a middle ground between going all out and avoidance, positioned reduced engagement as an ordinary situated response rather than a fixed trait.

The high exit costs created by institutional constraints make it nearly impossible for athletes to simply walk away. This constraint does not derive from any formal rule that explicitly binds enrollment to training attendance. It works through something more routine: in most cases, athletes admitted through the SSA policy are still required to take football courses, and team coaches typically serve as the instructors for those courses, which gives them direct evaluative authority over athletes' grades. The coach-instructor dual role lifts the potential cost of withdrawing from training, from “the coach might criticize me” to “my course grade might suffer.” Training participation is thus indirectly embedded within the academic evaluation system. This is the real risk athletes weigh when they consider leaving the team entirely.

The core category, “Navigating Constrained Training Participation,” captures how athletes interpreted constraints, assessed the risks of action, and moved among forms of avoidance as status, academic obligations, bodily conditions, coach relationships, and perceived consequences changed. It integrates constrained agency with the practical work of remaining in the team while limiting full compliance. The process varied in engagement intensity, behavioral visibility, need for justification, and institutional risk. These dimensions constitute gradient regulation, which is retained as a process property rather than a step-by-step developmental sequence.

P3's trajectory illustrates the dynamic character of this process. During his second year, conflict with a new coach was accompanied by recurrent psychological withdrawal and two consecutive weeks of unauthorized absence. Before the absence, he remained in the starting lineup because his playing ability continued to outweigh the coach's concerns about his training attitude; the psychological withdrawal and unauthorized absence were therefore classified as training avoidance while he still occupied a core playing position. He was moved to the bench only after returning from the two-week absence, which he understood as an informal sanction. After a period of self-directed training and a professional-club trial, his playing ability, relationship with the coach, and team position changed. He later assumed deputy captain and informal peer-coaching responsibilities and described greater responsibility toward training and the team. The case shows that avoidance strategies were neither fixed traits nor irreversible stages; they changed as status, relationships, competence, and perceived responsibility changed.

### 4.3 Action/interaction strategies

Four action/interaction strategies formed a flexible and context-dependent repertoire: Covert Energy Conservation, Psychological Defense, Legitimation Maneuvering, and Overt Resistance. Athletes could combine, skip, revisit, or abandon strategies as perceived risks, available justifications, bodily capacity, and relations with coaches changed. Their ordering below reflects increasing visibility and institutional risk, not a universal progression.

#### 4.3.1 Covert energy conservation

Covert Energy Conservation was the least visible and lowest-risk strategy in the repertoire. Athletes remained physically present while actively reducing physical effort or psychological investment. Its concealment allowed them to limit engagement while minimizing the likelihood of detection or sanction.

##### 4.3.1.1 Action attenuation

“On the field, if I want to take it easier, I just run a bit less, jog a little slower, put in less force, hang back in the queue.” (P12)

This captures the pattern of Action Attenuation, which takes on different forms depending on the type of training. In technical and tactical drills, the most common forms are reductions in speed and power.

During technical and tactical training, attenuation in speed and strength is the most common.

“During small-sided games you have to run back and forth, create space. Sometimes I just don't feel like making those extra runs. So I stand there and rest.” (P03)

When physical conditioning is conducted separately from technical and tactical training, physical conditioning becomes a high-incidence zone for action attenuation. P10 pointed out: “Strength class is just the default slacking-off class; if the coach isn't watching, you just sit there.” P11 further confirmed this: “Physical conditioning is the most tedious; when it rains and we move indoors, some people just sit in the corner, slacking off where nobody can see them.” Conducting action attenuation during strength and conditioning sessions has already formed a tacitly accepted consensus within the group.

Spatial attenuation is more concealed than attenuation in speed and strength. P03 provided a typical example: “When lining up, if there are two people ahead of me, I'll pretend to fall back, and when someone comes up behind, I just let them go first, so I can rest one more round.” This kind of attenuation occurs during the intervals when athletes take turns completing training drills; it is both difficult for the coach to detect and hardly causes any direct impact on teammates.

#### 4.3.2 Psychological defense

Psychological Defense describes a state in which participants' bodies remain on the training ground, but psychologically they have already withdrawn from the training situation. In this strategy, what student-athletes pull back is their emotional and cognitive investment. They are not daydreaming or drifting off. They are deliberately keeping their distance. This is an active behavior.

##### 4.3.2.1 Psychological withdrawal

Mind wandering has been shown to impair athlete performance in attention-demanding contexts (Li et al., 2024). P13 described the psychological disengagement at work: “Not being invested in training means your body shows up but your mind isn't on it. Like students zoning out in class, employees mo yu at work.” The term mo yu, an *in vivo* expression drawn from participants' own vocabulary, literally means “reducing effort” or “withholding effort.” In the everyday language of the team, it carries little moral charge. It functions less as a label and more as a description of common behavior.

“Nobody's watching you anyway. As long as you show up, that's enough.” (P12)

“Body present, mind absent: it's very common… but the key thing is, the body has to be there.” (P09)

The first comes from a marginal team member, the second from the captain. Both point to the same thing: in this strategy, participants pull their attention back. The cross-validation between two members occupying different positions in the team hierarchy confirms that Psychological Withdrawal is no secret within the team. It is a tacitly accepted, everyday state. As P15 put it: “It's just a normal state. Not particularly serious, not too slack either. You do what needs to be done. You don't go all out like in a match.” Training investment is held at a level that feels, to the athlete, “just enough to avoid being criticized.” As P04 put it: “When a match is coming up, even if you don't feel like training you grit your teeth and get it done. Coasting through training is just about not getting injured.” This, too, is regulation within a certain band. As competition draws near, student-athletes face a double pressure: on one side, the need to build fitness and form for the match; on the other, a body or mind already near its limit.

##### 4.3.2.2 Meaning erosion and sense of alienation

“If you never get any enjoyment out of training, never make progress, you just naturally fall away. You don't want to take part anymore.” (P07)

This captures Meaning Erosion and Sense of Alienation with precision. When participants keep showing up but cannot reach what they are aiming for, whether that is technical improvement, competitive opportunity, or simply enjoyment, their engagement shrinks. They grow distant from the act of training itself.

“Three years of college, and it's been like this the whole time. Training is so watered down. I feel like I'm regressing every day.” (P12)

“The main reason I don't want to train is that the training has no quality. I'm actually going backwards. Who'd want to train?” (P05)

Both accounts fit the same pattern.

From the participants' point of view, training is something you do in order to reach something. Even if that something is as modest as “getting the body moving” (P06). But when circumstances align as P04 described them, “The coach at our college team is pretty useless. No real content, doesn't know how to teach. Every session he just makes it up on the spot.” Neither the training itself nor the coach embedded in it can supply any meaning. And so the participant “naturally falls away.”

##### 4.3.2.3 Evaluative anxiety and error-avoidance

Football is a team sport through and through, and group evaluation and coach feedback are hardly rare occurrences within it. As P09 put it: “Not receiving the ball can be about lacking confidence. You‘re afraid you won't control it well and teammates or the coach will blame you. The pressure builds, and slowly you lose your confidence.” When participants fear the evaluation their mistakes will invite, anxiety sets in. That anxiety makes them doubt how they handle the ball. Confidence erodes. Avoidance follows.

“There are balls you could step up and challenge for, but you're afraid of making a mistake, afraid of getting beaten. So you don't dare step up. You just fall back conservatively.” (P14)

This, too, is a form of avoidance. The participant believes he is capable of the action, but fears that a mistake in executing it will bring criticism or blame, perhaps even affect the outcome. So he chooses to let it go. The point, then, is not that participants do not want to engage in training or show more initiative, pressing the ball, sprinting, and so on. It is that they fear being blamed for not doing it well enough.

Speaking as captain, P09 drew a sharper line: “Deliberately dodging, not proactively receiving the ball, not taking responsibility for your position on the pitch, that's being irresponsible to the team.” This judgment, interestingly, confirms the participants' concern from the reverse side. From their perspective, these behaviors are not about not caring for the team. They are about caring too much about where they stand and how they are seen within it. What this means is that evaluative anxiety-driven error-avoidance is liable to be misread, by an outside observer, as an attitude problem.

#### 4.3.3 Legitimation maneuvering

Legitimation Maneuvering involved translating a wish or need to reduce training into an account that coaches could recognize as legitimate. Compared with strategies that preserved physical presence, it exposed athletes to greater credibility risk if an explanation was challenged. Its defining feature was not its position in a fixed sequence, but the social work required to make absence institutionally legible.

Inter-Role Conflict created the need to prioritize incompatible obligations; Legitimation Maneuvering describes what athletes did with that conflict. Academic examinations, official meetings, and university duties could be invoked directly because coaches already recognized them as legitimate. Employment, family, and private obligations were less readily accepted and could therefore be omitted, reframed, or translated into a more institutionally recognizable account. The competing obligation is thus a causal condition, whereas invoking or repackaging it is an action/interaction strategy.

##### 4.3.3.1 Fabrication and packaging of justifications

“Sometimes it's really contradictory… you think about pretending to have a fever or a cold or something today and just not go. But then you think again… if you actually get to the field and there are especially many people asking for leave, the coach will be annoyed and think, how come you, this person, always have so many issues… If few people are asking for leave, maybe you just pretend you didn't bring your gear, show up empty-handed, without equipment. Tell the coach you have a headache. And then sometimes tell the coach, ‘Coach, I'm going to the gym to do some strength training instead.' Actually, most of the time in these situations, we just go back to the dorm and rest.” (P03)

This demonstrates that when student-athletes no longer want to stay on the field training, they often develop the idea of using “illness” as a reason for leave. However, due to uncertainty about the coach's attitude, they judge, based on the specific training situation of the day, which reason would be reasonable in that context, and then use that reasonable reason. For student-athletes, reasons of the type “all kinds of ways to get sick leave, like having a fever or something” (P05), “someone asked for leave claiming a family matter, but were actually going traveling” (P10), and “sometimes when you don't want to train, you just say you have a minor injury, then go sit on the sidelines and rest” (P15) are repeatable and templated, among which physical discomfort is the most common and safest reason.

P10 mentioned this situation: “Claiming that a family relative passed away, but then posting travel photos on social media that the teacher sees, that's definitely going to cause problems,” illustrating that such reasons carry the risk of being exposed. P13 later described, from his position as deputy captain, his attitude toward this behavior: “I can find out through other people whether a player's leave request is genuine. For example, leave for exams or referee training is understandable, but if someone simply doesn't want to train and makes up an excuse, if I find out, I'll definitely point it out.” This shows that the risk of a reason being exposed is not proportional to how cleverly it is crafted. The cost of being exposed is the coach's questioning of all future reasons from that athlete.

##### 4.3.3.2 Physiological boundaries and bodily politics

P5's account revealed two analytically distinct uses of injury. Achilles tendinitis constituted a genuine constraint on high-intensity training: “My body doesn't really allow such high-intensity training,” and his running range and performance declined when symptoms were present. Separately, he described occasions when a minor injury was invoked to justify rest when an athlete felt tired or unwilling to train: “Sometimes when you don't want to train, you just say you have a minor injury, then go sit on the sidelines and rest.” The analytical issue is not whether an injury was medically genuine, but how bodily discomfort could function both as a physical limitation and as a socially credible account for reduced participation.

“When women have their period, there's no way to take part in training. That's an important objective reason, not a subjective desire to dodge training.” (P07)

This reflects how physiological factors are gendered.

“For those with severe menstrual cramps, they can take those two days off. But the person still has to be present. Even if you're not training, you have to come to the pitch and help with support work.” (P10)

Menstruation occupies the clearest territory within physiological boundaries, backed as it is by biological fact. Yet P0's account reveals that even in this most indisputable case, athletes are still required to be there. The objectivity of the physiological reason is acknowledged. But it does not automatically convert into full absence.

P09, from his captain's vantage point, drew the basic line around physiological reasons: “Physical discomfort and injury, I can fully understand. That's common in competitive sport. But what I want to say is, when there's no injury, when your body is perfectly fine, and your training attitude is the problem, that's what I really care about.” P14 added a further distinction: “Injuries from matches and accidental injuries, those are understandable. But non-match injuries, like twisting your own ankle, hurting yourself, those are harder to accept.” What this shows is that even for objective physiological reasons, acceptability is graded by the source of the cause. Match collision injuries sit at the top. Chronic conditions sit somewhere in the middle. Self-inflicted injuries sit at the bottom. If a student-athlete wishes to invoke a physiological reason, they must position their bodily condition within this invisible grading system.

#### 4.3.4 Overt resistance

Overt Resistance comprised visible refusals that no longer depended on surface compliance or an institutionally acceptable justification. It included emotionally loaded displays, unauthorized absence, face-to-face refusal, and deliberate rejection of coaching directives. It carried the greatest visibility and institutional risk, but participants did not necessarily reach it by passing through all other strategies.

##### 4.3.4.1 Emotionally loaded negative display

P14 laid out a full picture of how negative emotions come out, moving from the mildest forms to the most extreme. It starts with words. Mutual cursing, blaming, foul language. The emotion comes out through speech, and even though there is a bit of aggression in it, training carries on. What happens next is a shift from words to the body. Someone kicks the ball flying with one foot. The signal changes. It is no longer just being in a bad mood. It says, “I am not cooperating.” And then, at the far end, someone simply leaves the field, turns their back on the session, and walks away. That is as far as it goes. Training stops for that person. What the coach might do about it later does not seem to matter anymore.

##### 4.3.4.2 Open defiance and unauthorized absence

Not every absence comes from a single emotional flare-up. P13 pointed to a case that took a different shape. During his sophomore year, after the coaching change, he simply stopped going to training for two straight weeks. No leave request, no explanation, just a clean break. “I figured I could not play well under his leadership anyway,” he said. This was not an outburst. It was a silent withdrawal that lasted and lasted. Not asking for leave was itself a signal. It said, plainly enough, “I am not going to explain to you anymore why I am not coming.” What it really signaled was that the athlete had stopped submitting to the coach's evaluative authority altogether.

There is actually an even more extreme form of face-to-face refusal. This is the furthest point that emotionally loaded negative display can reach. Indirect signals fall away, and what takes their place is a statement delivered face to face. There is no strategic wrapping on the words, no attempt to make them sound reasonable. At this moment, the relationship between athlete and coach gets pushed right to the edge of breaking. P10 did not actually quit in the end, because the coach talked them into staying.

A further form of overt resistance was the direct negation of the coach's authority during a match. P03 described it: “Sometimes when he's shouting from the sideline, we basically choose to pretend we can't hear him. We just make the adjustment ourselves.” The players did hear. They chose not to listen. Unlike walking off or failing to report, this behavior refuses the most fundamental power attached to the coach's role: the authority to direct tactics. It is less visible than booting the ball away. But it challenges the foundation of the coach-athlete relationship more thoroughly. The defining feature was enacted refusal: the players heard the instruction and deliberately suspended the coach's tactical authority.

### 4.4 Consequences

The strategies generated consequences across different time horizons. In the short term, reduced engagement could protect athletes from depletion, injury, evaluative exposure, or incompatible obligations. Over time, however, repeated avoidance could erode skill, belonging, and coach-athlete relationships. Consequences were not endpoints: long-term erosion could intensify marginalization and reshape subsequent strategy choices.

#### 4.4.1 Short-term protective consequences

In the short term, reducing effort, withdrawing attention, or obtaining an accepted absence created temporary relief. Athletes conserved energy, avoided public mistakes, protected injured or fatigued bodies, and made room for academic or livelihood obligations. These protective effects help explain why avoidance remained workable even when it conflicted with formal expectations of full engagement.

Short-term protection did not make the surrounding arrangements benign. It enabled athletes to remain in a system they could not easily exit while limiting immediate physical, psychological, or institutional costs.

#### 4.4.2 Long-term erosive consequences

Over time, sustained reductions in engagement could produce skill stagnation, deeper alienation from training, weaker relationships with teammates and coaches, and eventual withdrawal from sport. P11 observed that leaving the team could occur “by dorm unit,” indicating that erosion could also spread through everyday social networks and disrupt collective training.

Those who step outside the rules, however, do not step beyond their reach. P3's case reveals the cost of disruption: “Later when I went back to training, he wouldn't even give me my usual position. I was basically pinned to the bench for a while.” “The worst player on the team was getting game time. He just wouldn't let me play. It was punishment.” The coach's response was not formal institutional accountability. No demerit was recorded, no expulsion issued. It was personal sanction within the scope of training power: stripping the player of his position and a public shaming on the sideline. These informal sanctions could deepen marginalization and reproduce the conditions that had made avoidance meaningful, creating a recursive rather than strictly linear process.

## 5 Discussion

### 5.1 Navigating constrained training participation

This study developed a grounded theory of training avoidance among Chinese college football student-athletes. The theory is organized around the core category “Navigating Constrained Training Participation,” which explains how athletes interpreted constraints, assessed risk, and adjusted participation while remaining embedded in a system where exit was costly. Four action/interaction strategies—Covert Energy Conservation, Psychological Defense, Legitimation Maneuvering, and Overt Resistance—formed a flexible repertoire rather than a fixed sequence. They arose within Status Marginalization Pressure, Inter-Role Conflict, Informal Power Oppression, and Asymmetrical Rule Enforcement, and generated short-term protective and long-term erosive consequences.

Although the strategies identified here are rooted in Chinese college football, recognizable parallels appear in international literature. Covert disengagement and excuse-based absence have been described as reduced investment before dropout (Eliasson and Johansson, 2021); Psychological Withdrawal resonates with cognitive disengagement in avoidant coping (Nicholls and Polman, 2007); and Overt Resistance resembles behavioral markers associated with withdrawal decisions (Stavitz et al., 2025). The contribution is not that every behavior is entirely new, but that the study integrates them into a conditional process linking institutional pressures, constrained participation, flexible strategies, and consequences. Unlike the Athlete Engagement Questionnaire (AEQ; Lonsdale et al., 2007a,b), which assesses dimensions of athlete engagement, the present theory explains how engagement is actively navigated as constraints and anticipated consequences change. Gradient regulation remains useful only as a property describing variation in engagement intensity, behavioral visibility, need for justification, and institutional risk.

### 5.2 The pressure ecosystem: SDT and beyond

Self-Determination Theory (SDT) holds that the satisfaction of basic psychological needs for autonomy, competence, and relatedness is a necessary condition for maintaining intrinsic motivation and healthy behavioral regulation (Ryan and Deci, 2017). A lack of autonomy support is significantly associated with need frustration, declining engagement, and burnout (Mossman et al., 2024). Status Marginalization Pressure leaves student-athletes feeling that they are not valued members of the team, frustrating their need for relatedness. Yet the classic account of relatedness frustration (Ryan and Deci, 2017) and need thwarting (Bartholomew et al., 2011) focuses primarily on the direct psychological damage caused by active rejection or ostracism. What participants in this study described points to a more specific institutional condition. Athletes were not actively pushed away by coaches or teammates; they still occupied a position on the team. Their ongoing investment, however, received no response. The first author conceptualized this experience as “meaning erosion”: not the active thwarting of a need, but the institutional severing of the link between investment and return. This concept adds to the need-thwarting literature a pathway of frustration via institutional neglect rather than interpersonal rejection (Gunnell et al., 2013).

The four conditions do not operate in isolation but form an intertwined pressure ecosystem. Status Marginalization can diminish motivation and contribute to withdrawal (Eliasson and Johansson, 2021; Crane and Temple, 2015); Inter-Role Conflict can generate stress and burnout (Raedeke and Smith, 2001; Madigan et al., 2019); Informal Power Oppression can damage coach-athlete relationships (Jin et al., 2022; Jowett, 2005); and Asymmetrical Rule Enforcement can make the consequences of non-participation contingent on status and competitive value. Their intersection does not mechanically cause avoidance. Instead, it narrows the range of acceptable action and makes continued participation a problem athletes must navigate. This supports examining constellations of pressures rather than isolated antecedents (Crane and Temple, 2015), especially where exit is costly. Athletes' responses to informal power may also be understood as institutional helplessness: unlike classic learned helplessness (Seligman, 1972), it is rooted in identifiable arrangements of grading, selection, and authority rather than a generalized belief in uncontrollability.

### 5.3 Training avoidance as a structurally conditioned adaptation

The present study advances a non-moralizing rather than a neutral account of training avoidance. A non-moralizing position rejects the presumption that reduced engagement necessarily indicates deficient character or commitment; it does not deny that avoidance may have harmful consequences. Training avoidance was systemically produced in the sense that its conditions were embedded in institutional arrangements, but it was enacted through athletes' situated judgments and constrained agency. Its adaptive character was therefore temporally ambivalent: it could protect athletes from immediate depletion, injury, evaluative exposure, or role conflict while contributing to longer-term skill stagnation, alienation, and withdrawal.

The fear-avoidance model links avoidance to pain and fear of re-injury, often treating it as a response to be overcome (Dover and Amar, 2015), while avoidant coping research focuses primarily on psychological consequences for the individual athlete (Nicholls and Polman, 2007). The present study extends these accounts by showing that training avoidance also performs social and institutional functions. Covert Energy Conservation and Psychological Defense limited exposure while preserving formal presence; Legitimation Maneuvering made absence recognizable within institutional rules; and Overt Resistance challenged expectations more visibly. These were flexible responses to patterned conditions, not expressions of a single coping disposition or stages in a universal progression.

Training avoidance and athlete burnout (Madigan et al., 2019; Smith, 1986) may overlap but remain analytically distinct. Burnout is a multidimensional psychological syndrome, whereas training avoidance refers here to situated actions that reduce, redirect, conceal, justify, or resist expected engagement. P14 used burnout-related language to describe intense fatigue and loss of interest but later distinguished what he called a fatigue period from subjective avoidance. Other negative cases reinforced this boundary: P07 reported reluctance to wake for early training but engaged fully after attending, P16 described fatigue without a wish to avoid training, and P10 continued training during acute stress. Burnout-related states may therefore condition avoidance or emerge alongside it, but fatigue and reduced motivation are not sufficient to establish an avoidance strategy. Disengagement is similarly broader: it can describe declining identification or involvement, while training avoidance specifies how reduced participation is enacted within ongoing team membership.

Burnout may represent one pathway into avoidance, but avoidance also arose from unequal competitive opportunities, inter-role conflict, informal power, and asymmetrical rule enforcement. The definition developed here is non-moralizing rather than institutionally neutral. Participants used *in vivo* expressions such as mo yu and hua shui to describe ordinary reductions in effort without necessarily invoking character deficiency. These accounts support treating training avoidance as an independent behavioral descriptor while recognizing that its meaning and consequences are systemically conditioned.

### 5.4 Institutional asymmetry and cultural specificity

Conceptualizing training avoidance as a systemically conditioned process requires attention to sociocultural meaning. Chinese college student-athletes admitted through the SSA policy have long borne stereotypes of academic inadequacy (Chen and Ni, 2024), while being expected to satisfy competing training and coursework obligations. Reductions in engagement can therefore be stripped of context and attributed to personal attitude. The present framing shifts the analytical question from “What is wrong with this athlete?” to “How do institutional arrangements shape the ways this athlete can participate?” without denying athletes' capacity to judge, select, and revise their actions.

The identity ranking of “student first, athlete second” is not unique to China's SSA system. Identity conflicts among dual-career athletes are widespread across national sport systems, though their resolution varies with institutional arrangements (Stambulova and Wylleman, 2019). In the Chinese SSA context, this ranking forms a theoretically significant mirror image of the NCAA's “student-athlete” discourse. The NCAA deploys the term to safeguard the legitimacy of amateurism and to protect athletes' educational rights. Under the SSA system, the same identity ranking is mobilized by the institution to transfer the cost of academic-training conflict onto the individual athlete. Chinese college football should therefore not be treated as an isolated cultural exception, but as a highly visible case of constrained sport-education participation. The theory may be transferable to other systems in which scholarships, selection, educational progression, or access to resources make exit costly and distribute participation risks unequally.

Opaque evaluation within Informal Power Oppression, error-avoidance misread as an attitude problem, and Asymmetrical Rule Enforcement share a problem of institutional asymmetry. Athletes could not always predict how performance, absence, or disagreement would be evaluated. Navigating constrained participation captures their practical response to this uncertainty: they calibrated how much to invest, what to reveal, which explanation to offer, and when visible resistance became worth the risk.

Emotional contagion (Campo et al., 2019) travels along affective networks. The spread of negative behavior in this study presents itself as two distinguishable yet potentially interacting pathways. Collective withdrawal by dormitory unit travels along social-structural networks, mediated by daily interaction and shared institutional location. The interaction between these two pathways has yet to be fully explored in the present data, but it offers a direction for future research. It is worth noting that, in the context of Chinese authoritarian coaching, the “norms” governing emotional expression may be more than internalized team consensus; they may represent an institutional circumvention of the coach's evaluative power. Even though the quality of the coach-athlete relationship plays a critical role in athletes' sport experience and continued participation (Davis et al., 2019), when the coach also serves as the instructor of specialized courses and holds grading authority, communication ceases to be the “two-way strategic negotiation” that Davis and colleagues describe. It is compressed into the athlete's unilateral risk assessment.

### 5.5 Methodological reflections

P09 appears in the data in two different roles. As a participant, he described Psychological Withdrawal as a widespread, tacitly accepted everyday state. As team captain, he judged error-avoidance behavior as “being irresponsible to the team.” The tension between these two voices is not noise in the data. It is a microcosm of how training avoidance behavior gets misread within the institution: what an observer, even an insider, sees from the outside as an attitude problem is precisely what the participant experiences from the inside as evaluative anxiety. The captain identity gave P09 access to the institution's normative vocabulary; his participant identity gave him access to the real experiences of marginal team members. The distinctiveness of this structural position provides a form of *post hoc* validation for the study's theoretical sampling.

Across the four strategies, a theme of boundary blurriness runs consistently. In Covert Energy Conservation, athletes exploit the ambiguity of spatial position to conceal their attenuation of effort. In Psychological Defense, the line between absent-mindedness and deliberate distancing is, to an outside observer, impenetrable. In Legitimation Maneuvering, the boundary between chronic injury and subjective fatigue is blurred even within the athlete's own mind; at times, the athlete cannot cleanly separate the two. These blurred zones are distributed across the strategies, but they share a common function: it is precisely their blurriness that makes avoidance behavior difficult for the institution to pin down and sanction. The blur is not an analytical flaw. It is a constitutive feature of avoidance behavior and the very condition of its long-term survival within the institution. This cross-category property further supports the revised core category: athletes were not simply moving up or down a behavioral scale, but navigating ambiguous boundaries that preserved room for action under surveillance and unequal authority.

The non-moralizing and systemic definition developed here is closely tied to grounded theory methodology. A design that began with an instrument presupposing that avoidance equals dysfunction might have filtered out participant accounts that normalized reduced effort or distinguished it from giving up sport entirely. Open coding preserved these meanings, axial coding separated conditions from strategies, and selective coding required a core category capable of integrating agency, constraint, and consequences. This analytical progression led to Navigating Constrained Training Participation rather than retaining a purely behavioral continuum as the final theoretical explanation.

### 5.6 Limitations and future directions

This study has limitations. Its analytical focus was theory construction rather than testing the prevalence or predictive strength of particular pathways. The four causal conditions and their relationships with strategy selection require examination across institutions, sports, gendered environments, and levels of competitive status. Participants with captaincy, coaching, or informal leadership responsibilities occupied role-positioned perspectives that may have shaped how they evaluated teammates, even though their first-person athlete accounts were distinguished during analysis. Total years of football involvement and systematic training were not elicited uniformly and therefore could not be examined as quantitative background variables. Personal goal orientation, prior academy experience, injury, and the availability of credible exit options may also alter how athletes navigate participation. The model should not be generalized to voluntary recreational contexts where leaving carries little institutional cost, or to reductions in training caused solely by medically prescribed restriction.

The study intentionally limited its sample to student-athletes and did not include coaches or institutional administrators. This design prioritized depth of the athlete perspective over breadth of multi-stakeholder coverage, serving the research aim of constructing a conceptual framework of training avoidance behavior grounded in athletes' own accounts. The study therefore cannot address how coaches and administrators perceive, interpret, or respond to the avoidance behaviors athletes report, nor potential discrepancies between athlete narratives and institutional perspectives within the same training context. A grounded theory study of talent development among biathlon athletes that included athletes, coaches, technical directors, and parents demonstrated the distinctive value of multi-stakeholder research designs (Jørgensen et al., 2024). Future research could adopt a comparable design, incorporating coach and administrator perspectives, to examine how training avoidance behavior is perceived and evaluated differently across stakeholder groups.

### 5.7 Practical implications

The findings carry several implications for collegiate sport management practice. The most important is that training avoidance behavior should not be reduced to an attitude problem or moral failing. The student-athletes in this study were responding situationally to multiple environmental pressures under structural institutional constraints.

The practical implication is that interventions should target the sources of pressure rather than the surface behavior. What looks from the outside like “slacking off” or “lack of commitment” is often a rational response to binding constraints. An athlete who reduces effort during conditioning may be someone who has endured 3 years of selection neglect. An athlete who is absent from training may be someone caught in an asymmetry of institutional penalties in which missing training risks a failed course but failing to submit a thesis delays graduation. From this perspective, the most effective way to reduce training avoidance behavior is not to suppress it through stricter discipline, but to reduce its frequency and risk level by improving the institutional and interpersonal environment in which athletes operate.

More concretely, coaches and administrators can begin by distinguishing among the different forms of avoidance. Covert Energy Conservation and Psychological Defense are low-risk, internalized coping patterns. The signals they send are that unmet needs exist within the training context, needs that may involve competitive opportunities, recognition of competence, appropriate adjustments to training content, or simply some negotiable space in the training schedule. Reading these behaviors as signals rather than problems would allow coaches and administrators to intervene and adjust at an early stage, rather than waiting until avoidance becomes overt before responding reactively. Overt Resistance, for its part, signals that the relationship between athlete and environment has reached a breaking point. Rather than attempting to contain it through disciplinary means at that moment, it may be more productive to reflect on what institutional and interpersonal pressures pushed the athlete to that position.

At the institutional level, several directions for adjustment merit consideration. In training arrangements, more flexible time options and more transparent selection mechanisms could help alleviate the pressures arising from status marginalization and Inter-Role Conflict. In coach-athlete relations, introducing communication channels that allow athletes to express training needs and difficulties without fear of penalty could reduce the erosion of training engagement caused by informal power oppression. A gradual move toward understanding and accommodating diversity in training engagement, rather than treating full attendance or maximum effort as the sole criteria for evaluating athlete commitment, would also be a meaningful step.

If the reduction of avoidance behavior is achieved, however, at the cost of intensifying athlete stress and depletion, such as by using stricter disciplinary constraints to force athletes to maintain high-intensity engagement when they are already physically and psychologically overloaded, then such a “reduction” may itself generate more serious consequences: burnout, injury, or complete dropout. The practical significance of the systemic, non-moralizing definition of training avoidance proposed in this study lies precisely here: it invites coaches and administrators to treat avoidance behavior first and foremost as a source of information about athlete states, not as an enemy to be eliminated, and to make more targeted, more humane management decisions on that basis.

The study thus also points to a paradox worth heeding in practice: reducing training avoidance should not itself become the goal.

### 5.8 Conclusion

Training avoidance should be understood neither as an inherently neutral behavior nor as evidence of individual moral failure. It is a systemically produced and structurally conditioned process of adaptation through which athletes navigate constrained participation. Student-athletes remained formally located within the team while adjusting physical effort, psychological investment, justification, and visible compliance in response to costly exit and unequal institutional conditions. Gradient regulation describes variation within this process; the central theoretical contribution lies in explaining how athletes exercise constrained agency through a flexible repertoire of strategies. Adaptation does not imply that the behavior is beneficial: short-term self-protection may coexist with or develop into long-term erosion of skill, belonging, and participation.

## Data Availability

The raw interview data are not publicly available because they contain sensitive personal information and sharing is not permitted under the conditions of the institutional ethics exemption and participants' informed consent.

## Supplementary material

Supplementary Table S1–S6 Tables S1-S6 presenting interview-guide development, representative coding chains, category integration, and negative-case refinement.

## References

[B1] Bartholomew K. J. Ntoumanis N. Ryan R. M. Thøgersen-Ntoumani C. (2011). Psychological need thwarting in the sport context: assessing the darker side of athletic experience. J. Sport Exerc. Psychol. 33, 75–102. doi: 10.1123/jsep.33.1.75 21451172

[B2] Battaglia A. Kerr G. Tamminen K. (2024). The dropout from youth sport crisis: not as simple as it appears. Kinesiol. Rev. 13, 345–356. doi: 10.1123/kr.2023-0024

[B3] Battaglia A. Kerr G. Tamminen K. A. (2022). A grounded theory of the influences affecting youth sport experiences and withdrawal patterns. J. Appl. Sport Psychol. 34, 780–802. doi: 10.1080/10413200.2021.1872732

[B4] Campo M. Mackie D. M. Sanchez X. (2019). Emotions in group sports: a narrative review from a social identity perspective. Front. Psychol. 10:666. doi: 10.3389/fpsyg.2019.00666 30984077 PMC6450421

[B5] Chen J. Ni W. (2024). ‘Do I need to reinvent myself?' Stigmatization of sport-related identities of Chinese students. Front. Psychol. 15:1386796. doi: 10.3389/fpsyg.2024.1386796 39717467 PMC11663884

[B6] Corbin J. Strauss A. (2015). Basics of Qualitative Research: Techniques and Procedures for Developing Grounded Theory, 4th Edn. Thousand Oaks, CA: SAGE.

[B7] Crane J. Temple V. A. (2015). A systematic review of dropout from organized sport among children and youth. Eur. Phys. Educ. Rev. 21, 114–131. doi: 10.1177/1356336X14555294

[B8] Davis L. Jowett S. Tafvelin S. (2019). Communication strategies: the fuel for quality coach-athlete relationships and athlete satisfaction. Front. Psychol. 10:2156. doi: 10.3389/fpsyg.2019.02156 31607989 PMC6770846

[B9] Dover G. Amar V. (2015). Development and validation of the Athlete Fear Avoidance Questionnaire. J. Athl. Train. 50, 634–642. doi: 10.4085/1062-6050-49.3.75 25793458 PMC4527448

[B10] Dwyer S. C. Buckle J. L. (2009). The space between: on being an insider-outsider in qualitative research. Int. J. Qual. Methods. 8, 54–63. doi: 10.1177/160940690900800105

[B11] Eliasson I. Johansson A. (2021). The disengagement process among young athletes when withdrawing from sport: a new research approach. Int. Rev. Sociol. Sport. 56, 537–557. doi: 10.1177/1012690219899614

[B12] Ellis C. (2007). Telling secrets, revealing lives: relational ethics in research with intimate others. Qual. Inq. 13, 3–29. doi: 10.1177/1077800406294947

[B13] Etherington K. (2007). Ethical research in reflexive relationships. Qual. Inq. 13, 599–616. doi: 10.1177/1077800407301175

[B14] General Administration of Sport of China Ministry of Education. (2024). Administrative Measures for the Enrollment of Sports Training and Martial Arts and Traditional Sports Majors in Ordinary Institutions of Higher Education. Beijing: General Administration of Sport of China.

[B15] Glaser B. G. Strauss A. L. (1967). The Discovery of Grounded Theory: Strategies for Qualitative Research. Chicago, IL: Aldine. doi: 10.1097/00006199-196807000-00014

[B16] Gunnell K. E. Crocker P. R. E. Wilson P. M. Mack D. E. Zumbo B. D. (2013). Psychological need satisfaction and thwarting: a test of Basic Psychological Needs Theory in physical activity contexts. Psychol. Sport Exerc. 14, 599–607. doi: 10.1016/j.psychsport.2013.03.007

[B17] Jin H. Kim S. Love A. Jin Y. Zhao J. (2022). Effects of leadership style on coach-athlete relationship, athletes' motivations, and athlete satisfaction. Front. Psychol. 13:1012953. doi: 10.3389/fpsyg.2022.1012953 36578680 PMC9791096

[B18] Jørgensen H. Mosewich A. D. McHugh T. L. F. Holt N. L. A. (2024). A grounded theory of personal development in high-performance sport environments. Psychol. Sport Exerc. 71:102568. doi: 10.1016/j.psychsport.2023.102568 38000779

[B19] Jowett S. (2005). The coach-athlete partnership. Psychologist 18, 412–415. doi: 10.1037/t80377-000

[B20] Kelly L. M. Cordeiro M. (2020). Three principles of pragmatism for research on organizational processes. Methodol. Innov. 13:2059799120937242. doi: 10.1177/2059799120937242

[B21] Li G. Bian H. Li Y. (2022). 困境与突破: 新时代体教融合背景下高校体育发展策略研究 [Dilemma and breakthrough: university sport development strategies under sport-education integration in the new era]. 西安体育学院学 [*J. Xi'an Phys. Educ. Univ*.] 39, 633-640. [in Chinese]. doi: 10.16063/j.cnki.issn1001-747x.2022.05.014

[B22] Li J. Li C. Xue S. He Y. (2024). Mind wandering is not always harmful in sports: the role of its content. Front. Psychol. 15:1348893. doi: 10.3389/fpsyg.2024.1348893 39171231 PMC11336697

[B23] Li N. (2024). 高校高水平运动队发展的本质要求与治理逻辑 [The essential requirements and governance logic of high-level university sports team development]. 北京体育大学学报 [*J. Beijing Sport Univ*.]. 47, 47-58. [in Chinese]. doi: 10.19582/j.cnki.11-3785/g8.2024.08.005

[B24] Lonsdale C. Hodge K. Jackson S. A. (2007a). Athlete engagement: II. Development and initial validation of the Athlete Engagement Questionnaire. Int. J. Sport Psychol. 38, 471-492. doi: 10.1037/t50268-000

[B25] Lonsdale C. Hodge K. Raedeke T. D. (2007b). Athlete engagement: I. A qualitative investigation of relevance and dimensions. Int. J. Sport Psychol. 38, 451-470.

[B26] Madigan D. J. Gustafsson H. Smith A. Raedeke T. D. Hill A. P. (2019). The BASES expert statement on burnout in sport. Sport Exerc Sci. 61, 6-7.

[B27] Mossman L. H. Slemp G. R. Lewis K. J. Colla R. H. O'Halloran P. (2024). Autonomy support in sport and exercise settings: a systematic review and meta-analysis. Int. Rev. Sport Exerc. Psychol. 17, 540-563. doi: 10.1080/1750984X.2022.2031252

[B28] Nicholls A. R. Polman R. C. J. (2007). Coping in sport: a systematic review. J. Sports Sci. 25, 11-31. doi: 10.1080/02640410600630654 17127578

[B29] Raedeke T. D. Smith A. L. (2001). Development and preliminary validation of an athlete burnout measure. J. Sport Exerc. Psychol. 23, 281-306. doi: 10.1123/jsep.23.4.281 28682196

[B30] Rubin H. J. Rubin I. S. (2012). Qualitative Interviewing: The Art of Hearing Data, 3rd Edn. Thousand Oaks, CA: SAGE.

[B31] Ryan R. M. Deci E. L. (2017). Self-determination Theory: Basic Psychological Needs in Motivation, Development, and Wellness. New York, NY: Guilford Press. doi: 10.1521/978.14625/28806

[B32] Seligman M. E. P. (1972). Learned helplessness. Annu. Rev. Med. 23, 407-412. doi: 10.1146/annurev.me.23.020172.002203 4566487

[B33] Shi G. Liu D. Zhou M. Miao L. (2024). 深化体教融合背景下高校高水平运动队建设的时代价值、现实困境与实践进路 [The era value, realistic dilemma, and practical approach of constructing high-level university sports teams under the background of deepening sport-education sintegration]. 体育学研 [*J. Sport Stud*.] 38, 34-43. [in Chinese]. doi: 10.15877/j.cnki.nsic.20250126.001

[B34] Smith R. E. (1986). Toward a cognitive-affective model of athletic burnout. J. Sport Psychol. 8, 36-50. doi: 10.1123/jsp.8.1.36

[B35] Stambulova N. B. Wylleman P. (2019). Psychology of athletes' dual careers: a state-of-the-art critical review of the European discourse. Psychol. Sport Exerc. 42, 74-88. doi: 10.1016/j.psychsport.2018.11.013

[B36] Stavitz J. Porcelli R. Block-Lerner J. Marks D. R. Katzman H. (2025). Burnout, identity loss and institutional gaps: a qualitative examination of sport discontinuation among NCAA Division III athletes. Sports 13:116. doi: 10.3390/sports13040116 40278742 PMC12031138

[B37] Taylor J. (2011). The intimate insider: negotiating the ethics of friendship when doing insider research. Qual. Res. 11, 3-22. doi: 10.1177/1468794110384447

[B38] Yang G. (2022). 体教融合背景下我国高校高水平运动队建设: 历史考察、经验凝练与优化策略 [Construction of high-level university sports teams in China under the background of sport-education integration: historical review, experience distillation, and optimization strategies]. 北京体育大学学 [*J. Beijing Sport Univ*.] 45, 33-46. [in Chinese].

