# Supplementary material for: Navigating constrained training participation: a grounded theory of training avoidance among Chinese college football student-athletes
Source: Front Psychol. 2026 Aug 3;17:1904154. doi: 10.3389/fpsyg.2026.1904154 (PMC13488452; doi:10.3389/fpsyg.2026.1904154)
Supplement: Supplementary file 1 [file Data_Sheet_1.pdf]

Supplementary Material

Navigating Constrained Training Participation: A Grounded Theory of Training Avoidance Among Chinese College Football Student-Athletes

Table S1. Consolidated stages of interview-guide development

Sampling stage	Core focus	Key example questions/probes	Rationale and reflexive revision
Phase 1 (P01-P05)<br>Consolidated exploratory stage	Concrete participation episodes; effort reduction; attendance; psychological presence; preliminary variation in reasons and responses.	"When you are physically present but mentally checked out, what specific actions do you take?" "Can you describe an absence that you considered justified?" An early prompt also asked about a "tipping point" for no longer hiding avoidance.	Early sensitizing prompts elicited concrete episodes. Reflexive memoing identified that the 'tipping point/stop hiding' wording could presuppose concealment and sequential progression; those prompts were removed and were not used as analytic categories.
Phase 2 (P06-P14)<br>Consolidated narrative and theoretical-sampling stage	Narrative reconstruction without preset categories; psychological boundaries and self-explanation; changes over time; team status; academic, livelihood, bodily, and coach-related conditions; group dynamics.	"Can you describe one time you attended training but felt you were not truly engaged-what happened and what were you feeling?" "How do you distinguish an acceptable absence from an unacceptable one?" "How has your approach to training changed over time?"	Preset dimensional categories and morally loaded wording were removed. Questions followed the sequence of concrete episode, interpretation, conditions, action, and consequence. Emerging differences in team status, role obligations, informal authority, and rule enforcement informed subsequent sampling.
Phase 3 (P15-P16)<br>Discriminant and boundary-testing stage	Negative cases; fatigue without avoidance; status change without a corresponding change in training orientation; category boundaries; consequence and feedback relationships.	"Can you describe a time when you felt physically exhausted but still participated fully?" "Did a change in team position alter how you approached training?" "What would count as strain or fatigue without training avoidance?"	As preliminary saturation was approached, interviews were narrowed for discriminant sampling and category-boundary testing. The final interviews refined the distinctions between fatigue and enacted avoidance and between status change and engagement regulation before saturation was confirmed.

Note. Table S1 presents three consolidated stages rather than every minor adjustment. Within these stages, the guide was revised three times in Phase 1 and three times in Phase 2, as reported in the manuscript. Newly emerging concepts informed subsequent data collection in accordance with Straussian grounded theory (1). Full guides are available from the corresponding author upon reasonable request, subject to ethical approval and confidentiality safeguards.

Table S2. Representative coding chains for causal conditions (12 chains)

Main category	Subcategory/property	Initial code	Participant	Supporting quotation
Status Marginalization Pressure	Meaningless participation	No playing time or recognized position	P09	"They don't feel valued in the team. No playing time, no position. Training just feels pointless, and after a while they stop showing up."
Status Marginalization Pressure	Lack of involvement	Persistent exclusion from official matches	P01	"Some guys train for ages and still never make it into an official match. Zero sense of involvement. Of course a lot of them want to leave."
Status Marginalization Pressure	Progressive disengagement	Substitutes giving up after continued exclusion	P13	"A lot of substitute players never get a chance to play. Their performance gets worse and worse. Gradually they stop participating. They give up on themselves. Let themselves go."
Inter-Role Conflict	Academic-training overload	Back-to-back specialized classes and team sessions	P05	"Specialized football class in the morning, team training in the afternoon... add it up, that's eight or nine sessions a week."
Inter-Role Conflict	Thesis-training conflict	Prioritizing a high-penalty academic obligation	P07	"My master's thesis draft was due. There was no way I could juggle the research workload and the intensive training. So I just let go of all the training after that."
Inter-Role Conflict	Livelihood-training conflict	Choosing paid work over training	P08	"Taking private coaching jobs outside, substitute teaching, stuff like that, is about making money... whichever one you think brings you greater benefit, whichever one helps you more, that's what you'll choose."
Informal Power Oppression	Coach-instructor evaluative power	Threatening course failure and restricting competition	P05	"The coach threatened us with failing grades, wouldn't let us go out for high-level competitions, held onto us like private property."
Informal Power Oppression	Opaque authority	Exclusion before performance could be demonstrated	P12	"First day I got here, he wouldn't let me play. He hadn't even seen me on the court yet. I have no idea why."
Informal Power Oppression	Suppressed conflict and emotional diffusion	Covert resistance under costly open disagreement	P09	"They don't dare push back openly. So they engage in covert resistance, don't put in full effort at training, and it drags down the whole team's training quality."
Asymmetrical Rule Enforcement	Competitive-value exemption	Attendance rules relaxed for key players	P14	"There's an attendance system, but some key players just don't show up, and the coach says nothing, because the matches need them."
Asymmetrical Rule Enforcement	Marginal absence left unaddressed	Non-attendance produces different responses by status	P14	"The ones who play badly also don't show up, and gradually they just stop playing."
Asymmetrical Rule Enforcement	Differential disciplinary response	The same mistake receives unequal treatment	P02	"Same training mistake. With that other player the coach might say nothing at all. With me, he'd bring it up again and again, chewing me out."

Table S3. Representative coding chains for action/interaction strategies (20 chains)

Main category	Subcategory	Initial code	Participant	Supporting quotation
Covert Energy Conservation	Action Attenuation	Reducing running speed and force	P12	"On the field, if I want to take it easier, I just run a bit less, jog a little slower, put in less force, hang back in the queue."
Covert Energy Conservation	Action Attenuation	Avoiding additional runs	P03	"During small-sided games you have to run back and forth, create space. Sometimes I just don't feel like making those extra runs. So I stand there and rest."
Covert Energy Conservation	Action Attenuation	Reducing effort when unobserved	P10	"Strength class is just the default slacking-off class; if the coach isn't watching, you just sit there."
Covert Energy Conservation	Action Attenuation	Falling back in the queue to gain rest	P03	"When lining up, if there are two people ahead of me, I'll pretend to fall back, and when someone comes up behind, I just let them go first, so I can rest one more round."
Psychological Defense	Psychological Withdrawal	Body present, mind elsewhere	P13	"Not being invested in training means your body shows up but your mind isn't on it. Like students zoning out in class, employees mo yu at work."
Psychological Defense	Psychological Withdrawal	Attendance without psychological presence	P12	"Nobody's watching you anyway. As long as you show up, that's enough."
Psychological Defense	Psychological Withdrawal	Maintaining only an ordinary minimum	P15	"It's just a normal state. Not particularly serious, not too slack either. You do what needs to be done. You don't go all out like in a match."
Psychological Defense	Meaning Erosion and Sense of Alienation	Loss of enjoyment and perceived progress	P07	"If you never get any enjoyment out of training, never make progress, you just naturally fall away. You don't want to take part anymore."
Psychological Defense	Meaning Erosion and Sense of Alienation	Perceived regression through low-quality training	P12	"Three years of college, and it's been like this the whole time. Training is so watered down. I feel like I'm regressing every day."
Psychological Defense	Evaluative Anxiety and Error-Avoidance	Avoiding the ball because of anticipated blame	P09	"Not receiving the ball can be about lacking confidence. You're afraid you won't control it well and teammates or the coach will blame you."
Psychological Defense	Evaluative Anxiety and Error-Avoidance	Conservative retreat to avoid an observable mistake	P14	"There are balls you could step up and challenge for, but you're afraid of making a mistake, afraid of getting beaten. So you don't dare step up. You just fall back conservatively."
Legitimation Maneuvering	Fabrication and Packaging of Justifications	Selecting a contextually credible excuse	P03	"You might pretend you didn't bring your gear, show up empty-handed, without equipment. Tell the coach you have a headache."

Main category	Subcategory	Initial code	Participant	Supporting quotation
Legitimation Maneuvering	Fabrication and Packaging of Justifications	Using a templated illness account	P05	"All kinds of ways to get sick leave, like having a fever or something."
Legitimation Maneuvering	Physiological Boundaries and Bodily Politics	Injury as a genuine functional constraint	P15	"My body doesn't really allow such high-intensity training."
Legitimation Maneuvering	Physiological Boundaries and Bodily Politics	Minor injury invoked as a credible reason to rest	P15	"Sometimes when you don't want to train, you just say you have a minor injury, then go sit on the sidelines and rest."
Legitimation Maneuvering	Physiological Boundaries and Bodily Politics	Menstruation as an objective bodily boundary	P07	"When women have their period, there's no way to take part in training. That's an important objective reason, not a subjective desire to dodge training."
Overt Resistance	Emotionally Loaded Negative Display	Kicking the ball away in frustration	P14	"And some just kick the ball flying with one foot."
Overt Resistance	Emotionally Loaded Negative Display	Walking off the training field	P14	"They just walk off the field, turning around and leaving without training."
Overt Resistance	Open Defiance and Unauthorized Absence	Two consecutive weeks of unexplained absence	P13	"I figured I could not play well under his leadership anyway."
Overt Resistance	Open Defiance and Unauthorized Absence	Deliberately ignoring tactical instructions	P03	"Sometimes when he's shouting from the sideline, we basically choose to pretend we can't hear him. We just make the adjustment ourselves."

Table S4. Representative coding chains for consequences (4 chains)

Main category	Subcategory/property	Initial code	Participant	Supporting quotation
Short-Term Protective Consequences	Injury prevention	Reducing effort to remain available for competition	P04	"Coasting through training is just about not getting injured."
Short-Term Protective Consequences	Immediate relief	Using an accepted account to obtain rest	P03	"Actually, most of the time in these situations, we just go back to the dorm and rest."
Long-Term Erosive Consequences	Collective withdrawal	Team exit spreading through everyday social networks	P11	Leaving the team could occur "by dorm unit."
Long-Term Erosive Consequences	Informal sanction and renewed marginalization	Benching after unauthorized absence	P13	"Later when I went back to training, he wouldn't even give me my usual position. I was basically pinned to the bench for a while... It was punishment."

Note. Tables S2-S4 report 36 representative coding chains. They provide an auditable illustration of the movement from participant accounts to initial codes and integrated categories; they are not an exhaustive listing of all 73 initial codes.

Table S5. Category integration and process logic

Paradigm element	Integrated category	Analytical meaning	Relationship within the grounded theory
Causal conditions	Status Marginalization Pressure; Inter-Role Conflict; Informal Power Oppression; Asymmetrical Rule Enforcement	Conditions narrowed acceptable responses and made full participation, reduced participation, or exit consequential.	Conditions made the central phenomenon intelligible but did not mechanically cause avoidance.
Central phenomenon	Training Avoidance as Constrained Participation	Athletes reduced, redirected, concealed, justified, or resisted expected engagement while remaining formally attached to the sport-education system.	The phenomenon linked structurally conditioned pressures with athlete judgment and action.
Core category	Navigating Constrained Training Participation	Athletes interpreted constraints, assessed risk, selected or combined strategies, and revised participation as circumstances changed.	The core category integrates conditions, action/interaction strategies, and consequences through constrained agency.
Process property	Gradient regulation	Variation occurred in engagement intensity, behavioral visibility, need for justification, and institutional risk.	This is a dimensional property of the process, not a mandatory linear sequence or developmental stage model.
Action/interaction strategies	Covert Energy Conservation; Psychological Defense; Legitimation Maneuvering; Overt Resistance	Strategies could be combined, skipped, revisited, or abandoned as bodily capacity, obligations, status, and coach relations changed.	The repertoire connected interpretation of conditions with short- and long-term consequences.
Consequences and feedback	Short-Term Protective Consequences; Long-Term Erosive Consequences	Immediate relief could preserve continued membership; repeated avoidance could erode skill, belonging, relationships, and future opportunities.	Long-term erosion and sanctions could intensify marginalization and reshape later strategy choices, producing a recursive rather than strictly linear process.

Table S6. Negative cases and category-boundary refinement

Case	Apparent fit with an emerging explanation	Contrasting evidence	Boundary refined
P15	Moved from substitute status to a regular starting role.	General training orientation reportedly changed little after the status transition.	Improved status did not necessarily produce increased engagement; status was a condition, not a deterministic predictor.
P16	Reported fatigue under higher training loads.	Did not describe a wish to avoid training or an enacted avoidance strategy.	Fatigue alone was insufficient for coding training avoidance.
P07	Reported reluctance to wake for very early training.	Could participate fully after attending despite the initial reluctance.	Anticipatory reluctance was distinguished from enacted reduction, concealment, justification, or resistance.
P10	Reported acute stress during the training period.	Continued training rather than reducing or resisting expected participation.	Stress could condition experience without necessarily generating an avoidance strategy.

Supplementary Reference

1. Corbin J, Strauss A. Basics of qualitative research: techniques and procedures for developing grounded theory. 4th ed. Thousand Oaks, CA: SAGE (2015).
